# Congenital orbital teratoma presenting as a fungating keratinized mass in a resource-limited setting: a case report

**DOI:** 10.1186/s12886-026-04685-0

**Published:** 2026-02-20

**Authors:** Isa Salo Abdo, Dinksira Bekele Deneke, Metti Kuma Dida, Rezal Mohammed

**Affiliations:** 1 College of Health Science, Department of Pathology, Addis Ababa University, Addis Ababa, Ethiopia; 2 Department of Radiology, Yekatit 12 Hospital Medical College, Addis Ababa, Ethiopia; 3 College of Health Science, Department of Ophthalmology, Addis Ababa University, Addis Ababa, Ethiopia

**Keywords:** Congenital orbital teratoma, Orbital mass, Proptosis, Fungating

## Abstract

**Background:**

Orbital tumors present at birth are rare and pose significant diagnostic and management challenges, especially when they exhibit late and aggressive features such as fungation and keratinization. Early identification and intervention are crucial in preventing functional and cosmetic complications.

**Case Presentation:**

We report the case of a 9-month-old female infant who presented with a protruding left eye since birth. On physical examination, a large fungating mass with a keratinized surface occupying the left orbital region was noted. Detailed imaging and histopathological evaluation were performed to determine the nature of the mass. Management strategies and outcomes are discussed.

**Conclusion:**

This case highlights the importance of early recognition and multidisciplinary management of congenital orbital masses. Our findings contribute to the limited literature on congenital orbital neoplasms presenting with fungating features, underscoring the necessity for timely diagnosis and intervention.

## Introduction

Orbital masses in neonates and infants are exceedingly rare and can arise from a broad spectrum of benign and malignant etiologies [1, 2]. Orbital swelling at birth is particularly uncommon and may suggest underlying congenital neoplasia or vascular anomalies. Early clinical recognition is crucial, as delayed management can result in irreversible visual impairment, disfigurement, or damage to adjacent structures [3, 4].

In this report, we present a rare case of a congenital orbital mass manifesting with progressive proptosis and fungation, with prominent keratinization noted on physical examination. In this case, we aimed to emphasize the importance of early diagnosis, detailed histopathological evaluation, and prompt treatment in optimizing clinical outcomes.

## Case presentation

A 9 month old female infant from a rural part of Ethiopia was brought to our facility with a history of protrusion of the left eye that had been present since birth. The parents reported progressive enlargement of the mass over time, accompanied by intermittent mucopurulent discharge but no episodes of bleeding or pain. There was no history of trauma, prior surgical intervention, or other medical conditions. The patient’s prenatal and birth history was unremarkable, and there was no family history of similar conditions.

**Fig. 1:**
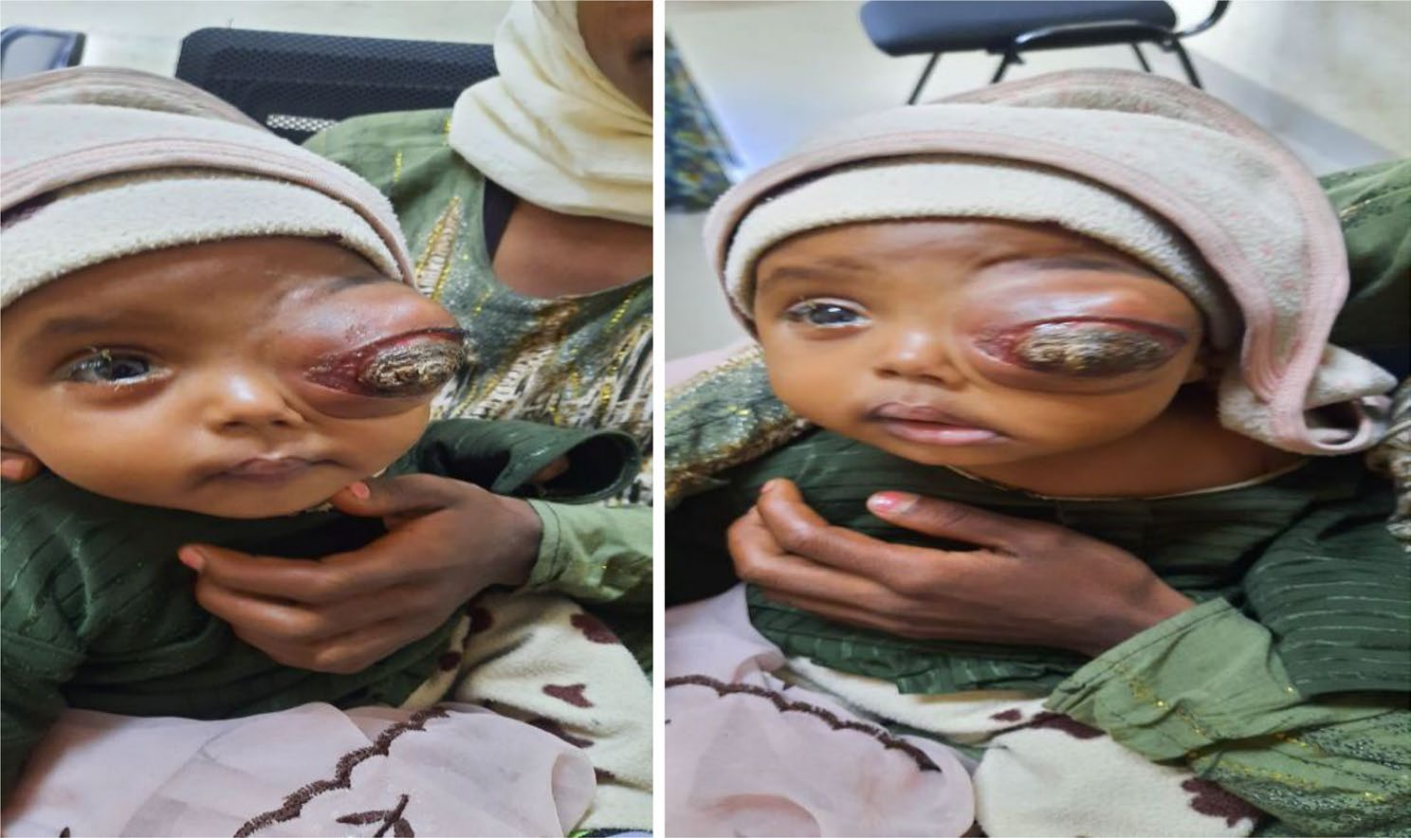
Clinical presentation of the patient demonstrating a large, fungating, keratinized mass occupying the left orbital region

On an ophthalmologic examination, a large fungating and keratinized mass occupied the left orbital region, resulting in marked proptosis, complete occlusion and forward protrusion of the left globe (Fig. 1**).** The surface of the mass appeared irregular, with areas of thickened keratin. Associated with stretched and erythematous changes in the overlying skin, no intact conjunctival structures were visualized. The right eye was normal, with preserved visual acuity and no abnormalities on fundoscopic examination. The laboratory tests results were within the normal range.

**Fig. 2:**
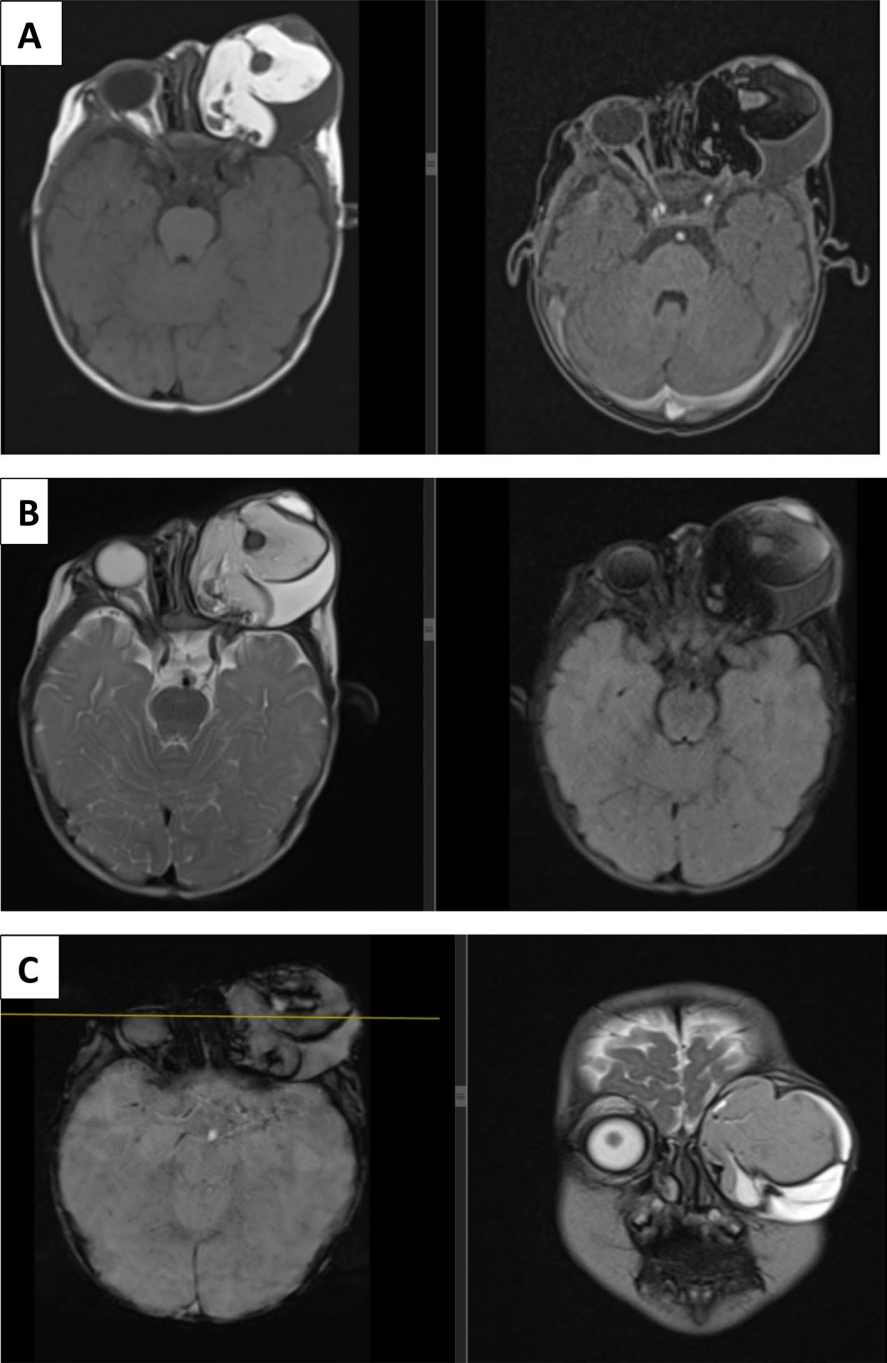
(**A**) T1 and T1 FS axial images showing heterogenous mass with a fat component in left orbital cavity. Septal smooth enhancement was observed. (**B**) T2 and FLAIR FS images showing heterogenous mass in left orbital cavity with cystic and fat component. (**C**) SWI and T2 coronal images showing the left orbital mass with blooming regions

Brain magnetic resonance imaging (MRI) with contrast revealed a large heterogeneous mass in the left orbital cavity (Fig. 2), measuring 5 × 5.7 × 5.3 cm. It has a fatty component (T1 hyperintense and suppression on T1 FS) and a cystic component (T2 hyperintense suppression on FLAIR). On GRE, multiple areas of blooming occur. In the postcontrast image, the septa and periphery of the mass showed smooth enhancement. The left eye globe was not observed separately. The mass compressed the ipsilateral ethmoid and maxillary sinuses. No intracranial extension was observed. The right orbital cavity contents were normal.

**Fig. 3:**
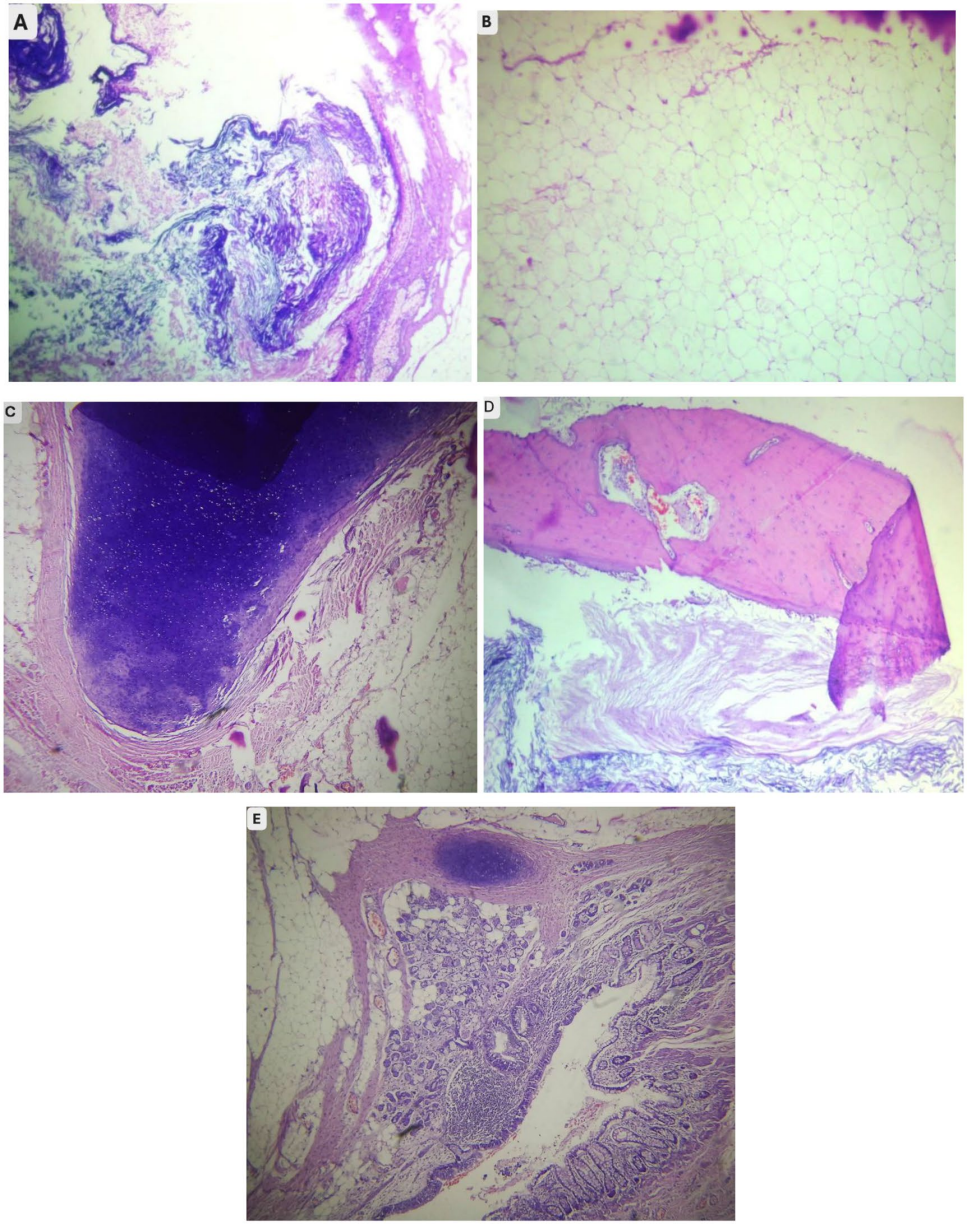
Hematoxylin and eosin Staining of Orbital Teratoma showing mature tissues from all three germ layers, including (**A**) squamous epithelium with keratin flakes, (**B**) Adipose tissue, (**C**) cartilage, (**D**) bone, and (**E**) glandular respiratory or gastrointestinal type epithelium

Orbital exenteration was performed under general anesthesia. Histopathological examination revealed a complex tumor composed of mature tissues derived from all three germ layers (Fig. 3). The ectodermal components included stratified squamous epithelium with abundant keratin flakes (A); the mesodermal elements consisted of adipose tissue, cartilage, and bone (images B-D); and the endodermal derivatives were represented by gastrointestinal-type and respiratory-type epithelia with associated mucous glands (E). These histologic findings were consistent with a diagnosis of mature teratoma.

Although teratoma’s are mostly benign, orbital exenteration was considered the most appropriate clinical management in this patient due to the tumor’s massive size and its secondary effects on ocular integrity. Preoperative clinical assessment confirmed that vision was **irreversibly lost**, with no identifiable light perception. Furthermore, the globe itself was found to be **structurally unsalvageable** due to the severe, chronic mass effect, which had resulted in extreme proptosis, stretching of the optic nerve, and loss of viable anatomical landmarks. Given the lack of visual potential and the inability to preserve a stable globe, exenteration was performed to achieve complete resection and facilitate prosthetic rehabilitation.

The postoperative recovery was unremarkable, with the surgical site healing well without any complications. The patient has been followed for the last 10 months post-surgery, during which time she has undergone regular clinical surveillance. In addition to clinical follow-up, she underwent a one-time supplement follow-up CT imaging study, which confirmed the complete absence of residual or recurrent tumor. At the most recent assessment, there was no evidence of recurrence.

## Discussion

Epidemiologically, congenital orbital teratomas are rare worldwide. Our review of the literature suggests that reports from the African continent remain infrequent (only three published case reports), highlighting the need for greater documentation to better understand the regional incidence and any unique presentation patterns in Africa.

Orbital teratomas are congenital tumors that typically present at or shortly after birth with progressive proptosis. The most common locations for teratomas are the gonads, sacrococcyx, retroperitoneum and mediastinum [5–7]. They are composed of tissues derived from one or more germ layers: the ectoderm, mesoderm, and endoderm [5, 7, 8].

Congenital orbital teratomas are generally benign and rarely they become malignant. Most orbital teratomas are benign, although their locally aggressive growth can cause significant orbital expansion, bony remodeling, and visual compromise if not promptly treated [9]. Moreover, if orbital teratoma are incompletely excised, recurrence or even malignant transformation may occur [10, 11].

In our case, the patient presented classic features of a congenital orbital teratoma, including unilateral proptosis noted since at birth [12].

The presence of keratinization and a fungating external appearance is unusual for orbital teratomas and may reflect secondary infection, ulceration, or surface breakdown due to prolonged mass effects.

Laboratory markers play a crucial role in both diagnosis and long-term surveillance in cases of congenital orbital teratoma. Alpha-fetoprotein (AFP) is one of the most commonly used markers. Although neonates naturally have high baseline levels, persistently elevated or rising AFP levels post-resection may indicate the presence of immature elements or a yolk sac component. These may indicate potential malignancy or recurrence.

Radiological imaging is essential in the assessment of orbital masses. CT and MRI help define the extent of the lesion, detect calcifications, differentiate cystic from solid components, and rule out intracranial extension [12]. In our patient, the imaging findings of an orbital mass with fat and cystic components suggested orbital teratoma, which was correlated with the histologic diagnosis.

Histopathology plays a crucial role in the definite diagnosis of orbital teratomas. In this patient, the histologic section revealed the characteristic features of a mature teratoma derived from all three germ layers [13], such as the squamous epithelium, cartilage, bone, adipose tissue, and glandular epithelium. No immature component identified.

The mainstay of treatment for orbital teratomas is complete surgical excision [14]. The choice of surgical approach depends on the tumor size, the involvement of adjacent structures, and the goal of preserving both cosmetic and functional outcomes. In recent years, globe preservation surgery has been more widely favored in certain patients to preserve the globe and promote normal orbital growth [9]. However, such conservative approaches are only viable when the globe is structurally intact and visual potential remains. In our case, the extreme chronic mass effect rendered the eye unsalvageable and vision irreversibly lost, necessitating exenteration.

Despite the need for aggressive surgical management, the prognosis for patients with mature orbital teratomas is generally favorable if complete excision is achieved. Recurrence is rare but mandates regular clinical and radiological follow-up.

This case highlights the importance of early diagnosis and intervention in congenital orbital tumors. Furthermore, the unusual presentation of a fungating keratinized mass underscores the diverse clinical manifestations that can be encountered in orbital teratomas.

## Conclusion

Congenital orbital teratomas, although rare, should be considered in the differential diagnosis of neonatal or infantile proptosis. A multidisciplinary approach involving detailed imaging, histopathological evaluation, and surgical management is essential for optimal outcomes. Early recognition and prompt intervention can prevent irreversible complications and improve both functional and cosmetic prognoses. Our case contributes to the limited body of literature on orbital teratomas, particularly those presenting as a fungating, keratinized masses.

## Data Availability

No datasets were generated or analysed during the current study.

## Abbreviations

CT: Computerized tomography scan
MRI: Magnetic resonance imaging scan
